# Indole-3-Carbinol (I3C) Protects the Heart From Ischemia/Reperfusion Injury by Inhibiting Oxidative Stress, Inflammation, and Cellular Apoptosis in Mice

**DOI:** 10.3389/fphar.2022.924174

**Published:** 2022-06-06

**Authors:** Qi Li, Boyu Xia, Jingjing Wu, Xiaomei Yuan, Xu Lu, Chao Huang, Hongcheng Gu, Koulong Zheng, Qingsheng You, Kun Liu

**Affiliations:** ^1^ Department of Cardiothoracic Surgery, Affiliated Hospital of Nantong University, Nantong, China; ^2^ Department of Cardiology, Suzhou Kowloon Hospital of Shanghai Jiaotong University School of Medicine, Suzhou, China; ^3^ Department of Cardiology, Sichuan Provincial People’s Hospital, University of Electronic Science and Technology of China, Chengdu, China; ^4^ Department of Pharmacology, School of Pharmacy, Nantong University, Nantong, China; ^5^ Medical College, Nantong University, Nantong, China; ^6^ Department of Cardiology, The Second Affiliated Hospital of Nantong University, Nantong, China

**Keywords:** indole-3-carbinol, ischemia/reperfusion, oxidative stress, inflammation, apoptosis

## Abstract

Strategies for treating myocardial ischemia in the clinic usually include re-canalization of the coronary arteries to restore blood supply to the myocardium. However, myocardial reperfusion insult often leads to oxidative stress and inflammation, which in turn leads to apoptosis and necrosis of myocardial cells, for which there are no standard treatment methods. The aim of this study was to determine the pharmacological effect of indole-3-carbinol (I3C), a phytochemical found in most cruciferous vegetables, in a mouse model of myocardial ischemia/reperfusion injury (MIRI). Our results showed that I3C pretreatment (100 mg/kg, once daily, i. p.) prevented the MIRI-induced increase in infarct size and serum creatine kinase (CK) and lactate dehydrogenase (LDH) in mice. I3C pretreatment also suppressed cardiac apoptosis in MIRI mice by increasing the expression levels of the anti-apoptotic protein Bcl-2 and decreasing the expression levels of several apoptotic proteins, including Bax, caspase-3, and caspase-9. In addition, I3C pretreatment was found to reduce the levels of parameters reflecting oxidative stress, such as dihydroethidium (DHE), malondialdehyde (MDA), reactive oxygen species (ROS), and nitric oxide (NO), while increasing the levels of parameters reflecting anti-oxidation, such as total antioxidant capacity (T-AOC) and glutathione (GSH), in MIRI-induced ischemic heart tissue. I3C pretreatment was also able to remarkably decrease the expression of tumor necrosis factor-α (TNF-α), interleukin-1β (IL-1β), and interleukin-6 (IL-6) mRNA in ischemic heart tissue. These results demonstrate that administration of I3C protects the heart from MIRI through its anti-apoptotic, antioxidant, and anti-inflammatory effects.

## Introduction

Ischemic heart disease is a major cause of mortality and morbidity in modern society (Hausenloy and Yellon, 2013). There is growing evidence that early and rapid thrombolytic therapy or direct percutaneous coronary intervention may help restore blood flow to the ischemic zone and may be developed as an effective strategy to reduce myocardial infarct size and improve the clinical outcome of cardiac ischemia/reperfusion (Yellon and Hausenloy, 2007). Paradoxically, the process of restoring blood flow in ischemic areas may also lead to irreversible death of myocardial cells, termed myocardial reperfusion injury (Piper et al., 1998), for which there are no standard treatments in the clinic. Therefore, it is importance to explore new approaches to mitigate myocardial ischemia/reperfusion injury (MIRI).

Scientific studies show that substances derived from natural plants, such as flavonoids and isoflavones, can reduce mortality from cancer and cardiovascular disease (Takachi et al., 2008). Indole-3-carbinol (I3C) is a small molecule derived from the genus Brassica (e.g., cabbage, cauliflower, broccoli, Brussels sprouts, and daikon) (Takada et al., 2005). Administration of I3C may have numbers of beneficial effects, including anti-inflammation, antioxidant, anti-virus, anti-angiogenesis, and promotion of tumor cell apoptosis (Kim and Milner, 2005; Mohammadi et al., 2017; Mohammadi et al., 2018). Treatment with I3C can also suppress the progression of inflammation-related lung cancer in mice by reducing the expression of tumor necrosis factor-α (TNF-α) and interleukin-6 (IL-6) (Song et al., 2015). In a high-fat diet (HFD) mouse model, I3C treatment was found to attenuate the HFD-induced increase in mRNA expression levels of TNF-α, IL-6, and interferon β (Choi et al., 2012). In a dorsal skinfold chamber model, I3C supplementation was found to suppress pro-inflammatory responses in the transverse muscle tissue of the dorsal skinfold compartment of mice by interfering with nuclear factor-κB (NF-κB) signaling (Ampofo et al., 2017).

I3C treatment may also have cardio-protective functions. For example, in a model of cardiac toxicity induced by doxorubicin (DOX) in mice, oral I3C administration was found to attenuate pathologically elevated reactive oxygen species (ROS) and lipid peroxidation in cardiac tissue (Hajra et al., 2018). In a rat model of isoproterenol-induced myocardial infarction, I3C treatment restores cardiac function by attenuating oxidative stress and cellular apoptosis (Ramakrishna and Krishnamurthy, 2022). In a heart failure model in mice, I3C supplementation stimulates mitochondrial oxidative capacity by activating peroxisome proliferator-activated receptor α (PPARα), peroxisome proliferator-activated receptor γ coactivator-1α (PGC-1α), or glucose transporter 4 (GLUT4)-mediated glucose uptake (Beauloye et al., 2011; Deng et al., 2014). I3C treatment may also improve myocardial uptake of glucose and fatty acids, thereby attenuating pressure overload-induced cardiac remodeling (Deng et al., 2014). In a previous study by Deng et al. (2013), I3C was found to inhibit cardiac remodeling through activation of 5′-adenosine monophosphate-activated protein kinase-α (AMPK-α) and extracellular signal-regulated kinase 1/2 (ERK1/2) (Deng et al., 2013), two molecules whose activation may protect the heart from ischemic injury and pressure overload-induced ventricular hypertrophy and dysfunction (Russell et al., 2004; Zhang et al., 2008). In addition, I3C treatment has been found to attenuate hyperoxia- and hypoxia-induced neonatal lung injury by preventing inflammation in a manner dependent on activation of the aryl hydrocarbon receptor (AhR) pathway (Popolo et al., 2017). However, to date, the direct effects of I3C supplementation on I/R-induced cardiac injury remain unclear. In the present study, we investigated this question using a model of MIRI in C57BL/6J mice. We also examined the effect of I3C supplementation on oxidative stress, cellular apoptosis, and pro-inflammatory responses in ischemic cardiac tissue in I/R-stimulated mice.

## Material and Methods

### Animals

Six-to eight-week-old C57BL6/J mice (male) were purchased from Beijing Vital River Laboratory Animal Technology Co., Ltd. (Beijing, China) and were kept five per cage under standard vivarium conditions (12-h light/dark cycle, lights on from 07:00 to 19:00, 23 ± 1 °C ambient temperature, 55 ± 10% relative humidity) for 1 week with free access to food and water. Animal experiments were approved by the University Animal Ethics Committee of Nantong University (Permit Number: 2110836) and were conducted in accordance with the internationally accepted guidelines for the use of animals in toxicology adopted by the Society of Toxicology in 1999.

### Materials

I3C, purchased from Med Chem Express (MCE, New Jersey, United States ), was dissolved in a solution containing dimethyl sulfoxide (DMSO) with PEG300, Tween-80, and saline (Galvao et al., 2014). The solution containing only DMSO with PEG300, Tween-80, and saline was used as the vehicle. The final concentration of DMSO is <0.05%. No toxic effect of DMSO was observed.

### Experimental Arrangement and Drug Treatment

After 1 week of acclimatization, mice were randomly divided into the Sham group, the I/R group, and the I/R + I3C group (*n* = 5 in each group). I3C was administrated intraperitoneally at a dosage of 100 mg/kg (Bailey et al., 2005) once daily for three consecutive days before surgery, on the day of surgery, and on the day after surgery.

### Establishment of the Myocardial I/R Model

For the myocardial I/R procedure (Lu et al., 2020), mice were anesthetized with inhaled isoflurane via an isoflurane delivery system (Yuyan Instruments, Shanghai, China). A small incision was then made in the left thorax of the mouse to expose the pectoralis major and minor. The pleura was punctured with eye in the fourth intercostal space. Then, the heart was manually pushed out with pressure on the upper back of the mouse and a slip knot was tied with a 6-0 polyamide six suture approximately 2 mm from the origin of the left anterior descending artery (LADA) to induce ischemia. Effective ligation was confirmed by the presence of a pale color in the anterior wall of the left ventricle. Thirty minutes later, the ligation was released to allow 24-h reperfusion of the LADA. The chest was then closed with a nylon suture. Mice in the sham groups underwent similar procedures except for closure of the LADA.

### Determination of Myocardial Infarct Size

Evans blue and 2, 3, 5-triphenyltetrazolium chloride (TTC) double staining was used to detect the changes in myocardial infarct size (Yu et al., 2018). Briefly, after 24 h of reperfusion, 1.5 ml of 1% Evans blue dye was injected into the left ventricle. Then, the heart was quickly excised, frozen at -20 °C, and cut into 2-mm-thick sections perpendicular to the long axis, resulting in four sections per heart. The prepared slides were incubated with 1% TTC at 37°Cfor 20 min, and tissue images were captured using a Canon 600D digital single-lens reflex camera (DSLR).

### Histopathological Examination

Histopathological examination of myocardial tissue from mice treated with vehicle, I/R, or I3C was performed by hematoxylin-eosin (HE) staining. Myocardial tissue sections were deparaffinized with xylene (5 min × 3 times), followed by incubation in hematoxylin solutions for 5 min. Then the sections were treated sequentially with hematoxylin differentiation solution, hematoxylin Scott tap bluing, and rinsing with tap water, followed by dehydration in 95% or 85% ethanol (5 min each). Finally, sections were mounted and examined with a light microscope (Nikon Eclipse E100, Nikon, Japan).

### Echocardiographic Analysis of Cardiac Function

After the end of reperfusion, cardiac functions of the isoflurane-anesthetized mice were examined with an *in vivo* ultrasound imaging system for small animal models (VEVO 2100, Visual Sonics, Toronto, Canada). M-mode tracings were recorded in a short-axis view of the left ventricle at the level of papillary muscle, and then the internal diameter of the left ventricular were measured at the end of diastole (LVID-d) and at the end of systole (LVID-s).

### Real-Time Reverse Transcriptase-PCR

Total RNA in the heart of mice treated with vehicle, I/R, or I3C was extracted using an RNeasy Mini Kit according to the manufacturer’s instructions (Qiagen, GmbH, Hilden, Germany). The first strand of cDNA was generated using a reverse transcription system (Promega, Madison, WI, United States ). Real-time PCR was performed using a reaction system containing 1× Faststart SYBR Green Master Mix (Roche Molecular Biochemicals), 2 μL diluted cDNA, 2 mM MgCl_2_, and 0.5 μM primers. Primers for *Tnf-α*, *Il-1β*, *Il-6* were cited as follows: *Tnf-α*, 5′-CTG​TGA​AGG​GAA​TGG​GTG​TT-3’ (F), 5′-GGT​CAC​TGT​CCC​AGC​ATC​TT-3’ (R); *Il-β*, 5′-TGG​AAA​AGC​GGT​TTG​TCT​TC-3’ (F), 5′-TAC​CAG​TTG​GGG​AAC​TCT​GC-3’ (R); *Il-6*: 5′-AGA​GAT​ACA​AAG​AAA​TGA​TGG​A-3’ (F), 5′-AGC​TAT​GGT​ACT​CCA​CAA​GAC​CA-3’ (R). PCR products were detected by observing the intensity of fluorescence of the double-stranded DNA binding dye SYBRGreen. Gene expression analysis was performed using the-ΔΔCt method.

### Isolation of Mitochondria

Isolation of mitochondria from equal weights of heart tissue was performed according to the instructions of the commercial mitochondria isolation kit (Beyotime, Shanghai, China). In brief, the hearts of the euthanized mice were rapidly removed and transferred to ice-cold PBS. The ischemic region of LV was minced and trypsinized in an ice bath. The collected heart tissue was homogenized in mitochondria isolation reagent A using a Potter-Elvehjem tissue grinder and centrifuged at 600 g for 5 min at 4°C. Mitochondria were recovered by further centrifugation of the supernatants at 11,000 g for 10 min, which were then suspended in 40 μL of mitochondrial storage fluid.

### ROS Assay

The mitochondrial ROS assay was performed with using a commercial ROS assay kit (Beyotime, Shanghai, China), using 20, 70-dichlorofluorescein diacetate (DCFH-DA) was used to monitor the changes in ROS level in purified mitochondria. The DCFH-DA could be cleaved to form DCFH in the presence of H_2_O_2_. The fluorescence intensity of DCFH was determined using the microplate reader (Synergy H1, BioTek, United States ) at an excitation of 488 nm and an emission of 525 nm. The final results were expressed as a percentage of the control.

### Measurements of Malondialdehyde (MDA), Glutathione (GSH), Nitric Oxide (NO), and Total Antioxidant Capacity (T-AOC)

MDA, GSH, NO, and T-AOC levels were measured in tissue homogenates of the left ventricles using the commercial MDA, GSH, NO, and T-AOC assay kits purchased from the Nanjing Jiancheng Bioengineering Institute (Nanjing, China). Results were normalized to total protein using the BCA method (Beyotime, Shanghai, China) and expressed as nmol MDA, nmol NO, nmol GSH, and units T-AOC mg protein in the homogenate, respectively.

### Measurement of Lactate Dehydrogenase (LDH) and Creatine Kinase (CK) Concentrations

Serum LDH and CK concentrations were determined using the appropriate commercial kits purchased from Nanjing Jiancheng Bioengineering Institute. Results were expressed as U/ml units.

### Myocardial Dihydroethidium (DHE) Staining

DHE staining was performed according to the manufacturer’s protocols. The 8 μm-thick frozen heart sections were first incubated with DHE solution (Servicebio, Wuhan, China) at 37°C for 30 min, and then DAPI was used to detect the nucleus. Staining images were viewed with a fluorescence microscope (Leica, Wetzlar, Germany).

### Myocardial TUNEL Staining

Cellular apoptosis was assessed using a One Step TUNEL Apoptosis Assay Kit (Beyotime, Shanghai, China). Harvested cardiac tissue was fixed with 4% paraformaldehyde (PFA), dehydrated, and embedded in OCT. The OCT-embeddings were cut into 8-μm-thick slices, which were then treated with PFA, permeabilized with 0.5% Triton X-100, and incubated in a working buffer at 37°C for 60 min according to the manufacturer’s instructions. Finally, the glycerol-sealed slides were analyzed using a fluorescence microscope (Leica, Wetzlar, Germany).

### Western Blot

Total proteins were extracted from the ischemic regions of cardiac tissue, and protein concentrations were determined by the BCA method (Beyotime, Shanghai, China). Protein samples, boiled at 95°C for 10 min, were separated in a 10% SDS-PAGE gel at 80 V for 1.5 h and transferred to nitrocellulose membranes (Merck Millipore, Darmstadt, Germany) at 260 mA for 90 min. After being blocked by nonfat milk (2 h) at room temperature, the nitrocellulose membranes were treated overnight at 4°C with the antibodies against caspase 3 (1:500), caspase 9 (1:500), and GAPDH (1:20000). On the second day, the primary antibodies were removed and the membranes were washed three times in TBST, followed by another incubation at room temperature for 2 h with the IRDye 680-labeled secondary antibody (1:3000–1:5000). Blot bands visualized using an Odyssey CLX Western blot detection system (LICOR, Nebraska, United States ) were quantified using ImageJ software.

### Statistical Analysis

All data were analyzed using one-way analysis of variance (ANOVA) or two-way ANOVA with repeated measures followed by Bonferroni multiple comparisons via GraphPad Prism 9 (Graphpad Software, CA, United States ). A value of *p* < 0.05 was considered statistically significant. Data are presented as mean ± standard error of the mean (SEM).

## Results

### Administration of I3C Prevented Myocardial Damage and Protected Cardiac Function in I/R Mice

**FIGURE 1:**
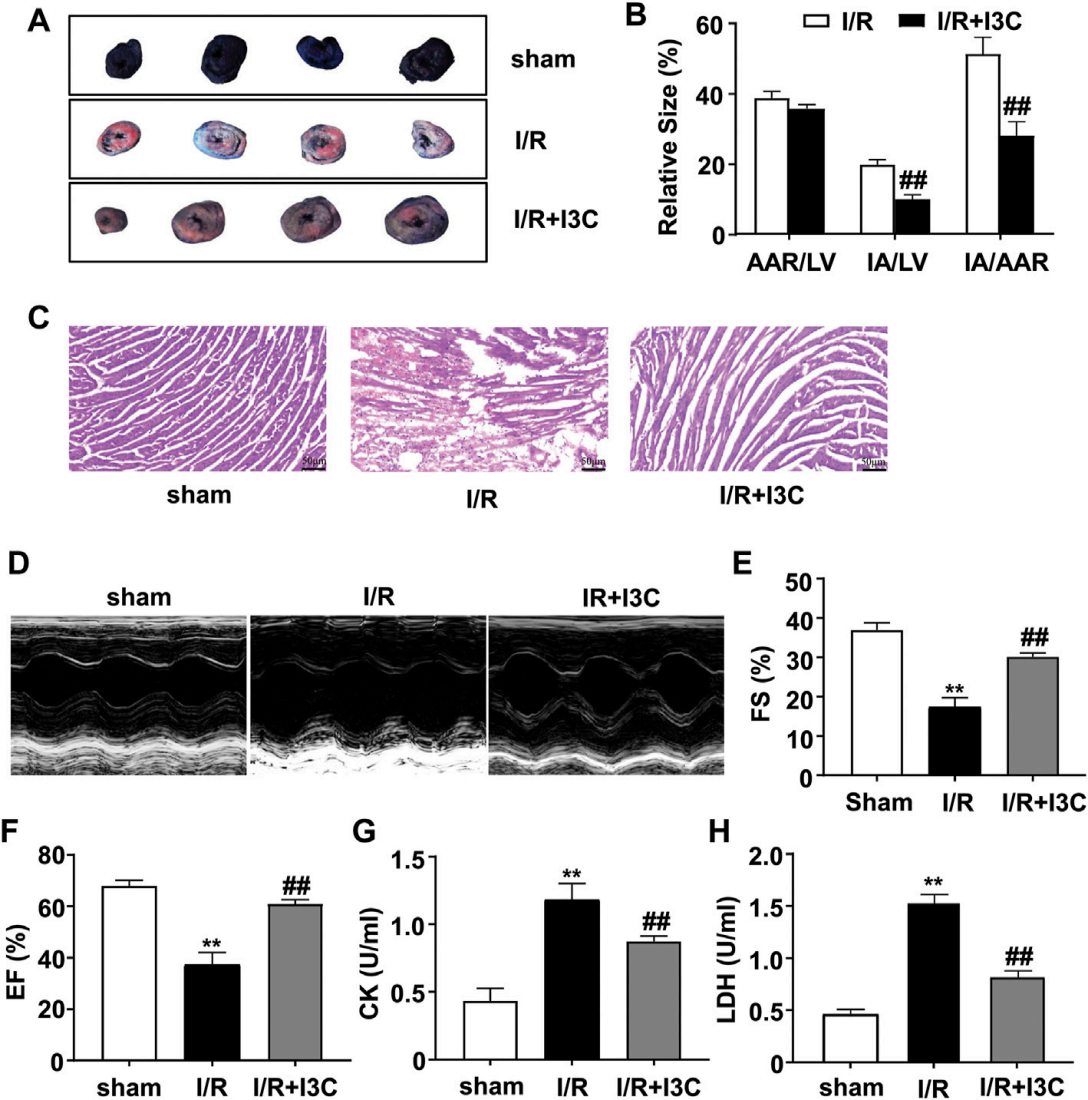
Effect of I3C on I/R-induced myocardial injury and cardiac function. **(A)** A representative photograph shows Evans blue/TTC double-staining in the heart of mice treated with or without I3C (100 mg/kg). The nonischemic area, which was not at risk, was stained blue, whereas the area at risk was red and the infarct area was pale. **(B)** Quantitative analysis showed infarct size of the heart 24 h after reperfusion in mice treated with or without I3C: I3C pretreatment reduced IA/LV in I/R mice from 19.73 to 9.90% and IA/AAR in I/R mice from 51.19 to 27.91% (n = 5, ##*p* < 0.01 vs. I/R); AAR, area at risk; LV, left ventricle; IA, infarct area. **(C)** Representative hematoxylin-eosin (HE) staining results showed I3C pretreatment reduced I/R-induced cardiac injury in mice. Scale bar: 50 μm. **(D)** Representative images show cardiac function determined by echocardiography. **(E,F)** Quantitative analysis showed that I3C pretreatment prevented the I/R-induced decrease in fractional shortening (FS, **(E)**) and ejection fraction (EF, **(F)**) values (n = 5, **p < 0.01 vs. sham treatment, ##*p* < 0.01 vs. I/R). **(G,H)** Quantitative analysis showed that I3C pretreatment prevented the I/R-induced increase in serum levels of creatine kinase (CK, **(G)**) and lactate dehydrogenase (LDH, **(H)**) (*n* = 5, **p < 0.01 vs. sham treatment, ##*p* < 0.01 vs. I/R). Data are expressed as mean ± SEM.

We first measured the changes in myocardial infarction areas in mice treated with vehicle, I/R, or I3C. The results showed that I3C pretreatment (100 mg/kg) significantly reduced the infarct area normalized to the left ventricle from 19.73 to 9.90% and the infarct area compared with the area at risk from 51.19 to 27.91% in I/R-treated mice (Figures 1A, B). The results of HE staining showed cardiac tissue impairment in the I/R mice, rescued by I3C pretreatment (Figure 1C), suggesting that I3C pretreatment may ameliorate the I/R-induced cardiac tissue damage. Echocardiography was then used to demonstrate the cardioprotective effect of I3C in I/R mice (Figure 1D). We found that I3C pretreatment significantly increased I/R-induced left ventricular ejection fraction (EF, Figure 1F, F_2,12_ = 130.10, *p* < 0.001) and fractional shortening (FS, Figure 1E, F_2,12_ = 156.20, *p* < 0.001) in mice. It was also found that I3C pretreatment caused a remarkable decrease in serum activities of CK (Figure 1G, F_2,12_ = 78.67, *p* < 0.001) and LDH (Figure 1H, F_2,12_ = 260.10, *p* < 0.001) in I/R mice.

### Administration of I3C Prevented the Attenuation of I/R-Induced Cellular Apoptosis in Ischemic Cardiac Tissues

**FIGURE 2:**
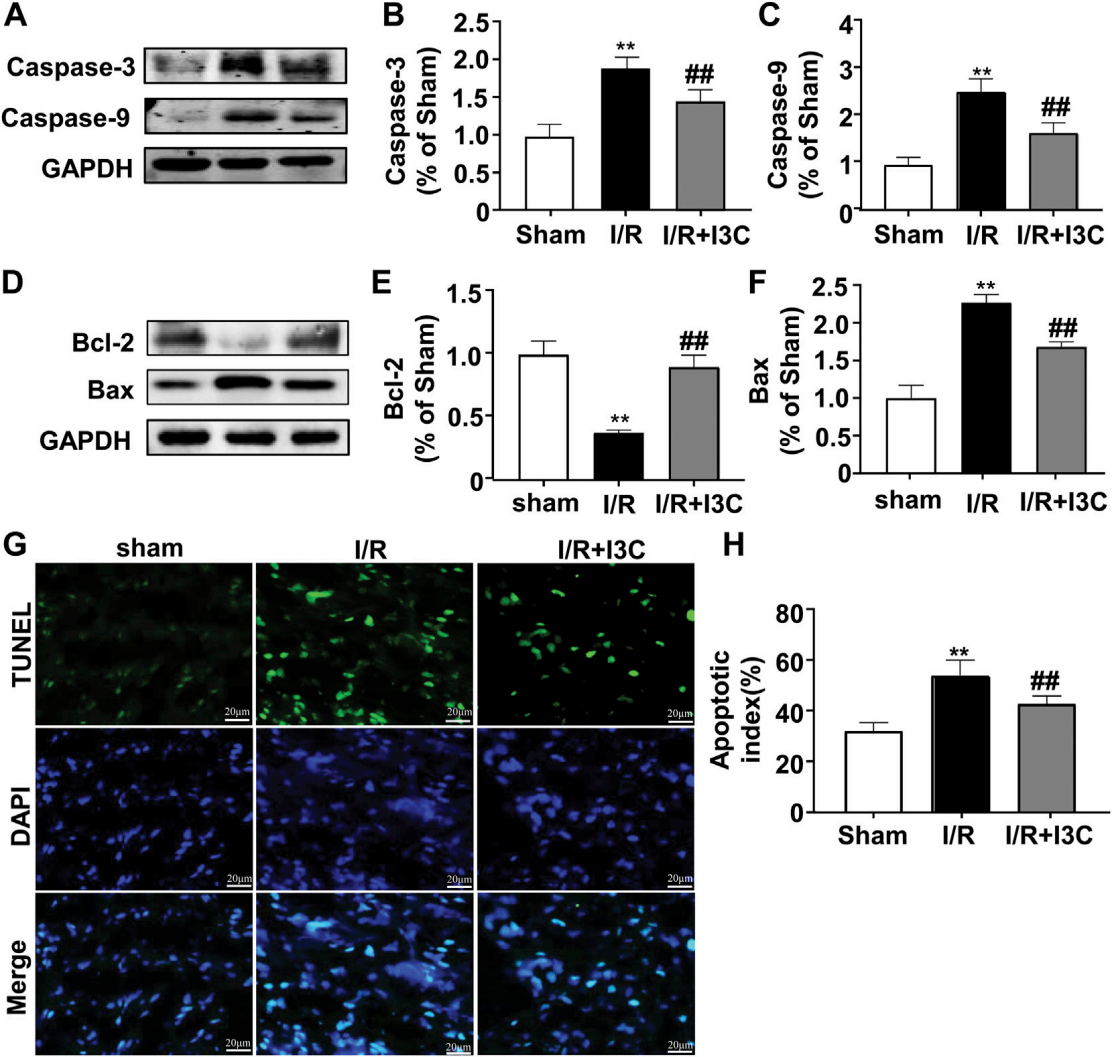
Effect of I3C on cardiac apoptosis induced by I/R. **(A–C)** Representative images and quantitative analysis showed that I3C pretreatment (100 mg/kg) prevented the I/R-induced increase in the expression levels of caspase-3 **(A,B)** and caspase-9 **(A,C)** in ischemic cardiac tissue (*n* = 5, **p < 0.01 vs. sham treatment, ##*p* < 0.01 vs. I/R) **(D–F)** Representative images and quantitative analysis showed that I3C pretreatment prevented the I/R-induced decrease in expression levels of Bcl-2 **(D,E)** and the I/R-induced increase in expression levels of Bax **(D,F)** in ischemic heart tissue (*n* = 5, **p < 0.01 vs. sham treatment, ##*p* < 0.01 vs. I/R). **(G,H)** Representative images **(G)** and quantitative analyses **(H)** showed that I3C pretreatment prevented the I/R-induced increase in the number of TUNEL-positive cells in ischemic heart tissue (n = 5, **p < 0.01 vs. sham treatment, ##*p* < 0.01 vs. I/R). Scale bar: 20 μm. Data are expressed as mean ± SEM.

Next, we examined the effect of I3C on I/R-induced cellular apoptosis in ischemic mouse cardiac tissues. For this purpose, the changes in the expression of caspase-3 and caspase-9 were detected in ischemic heart tissues from mice treated with I3C or I/R (Figures 2A, D). The results showed that I3C pretreatment (100 mg/kg) caused a remarkable decrease in the expression levels of the three pro-apoptotic proteins, Bax (Figure 2F, F_2,12_ = 136.40, *p* < 0.001), caspase-3 (Figure 2B, F_2,12_ = 41.95, *p* < 0.001), and caspase-9 (Figure 2C, F_2,12_ = 60.47, *p* < 0.001), and remarkable increases in the expression levels of the anti-apoptotic protein Bcl-2 (Figure 2E, F_2,12_ = 78.93, *p* < 0.001) (Figures 2A,D) in ischemic heart tissue from I/R-treated mice. TUNEL analysis showed the number of TUNEL-positive cells increased significantly in the ischemic heart tissue of I/R mice, and that this increase was attenuated by pretreatment with I3C (Figure 2H, F_2,12_ = 41.32, *p* < 0.001). These findings demonstrate that I3C pretreatment attenuates I/R-induced cellular apoptosis in the ischemic heart of mice.

### Administration of I3C Prevented I/R-Induced Oxidative Stress in the Heart of Mice

**FIGURE 3:**
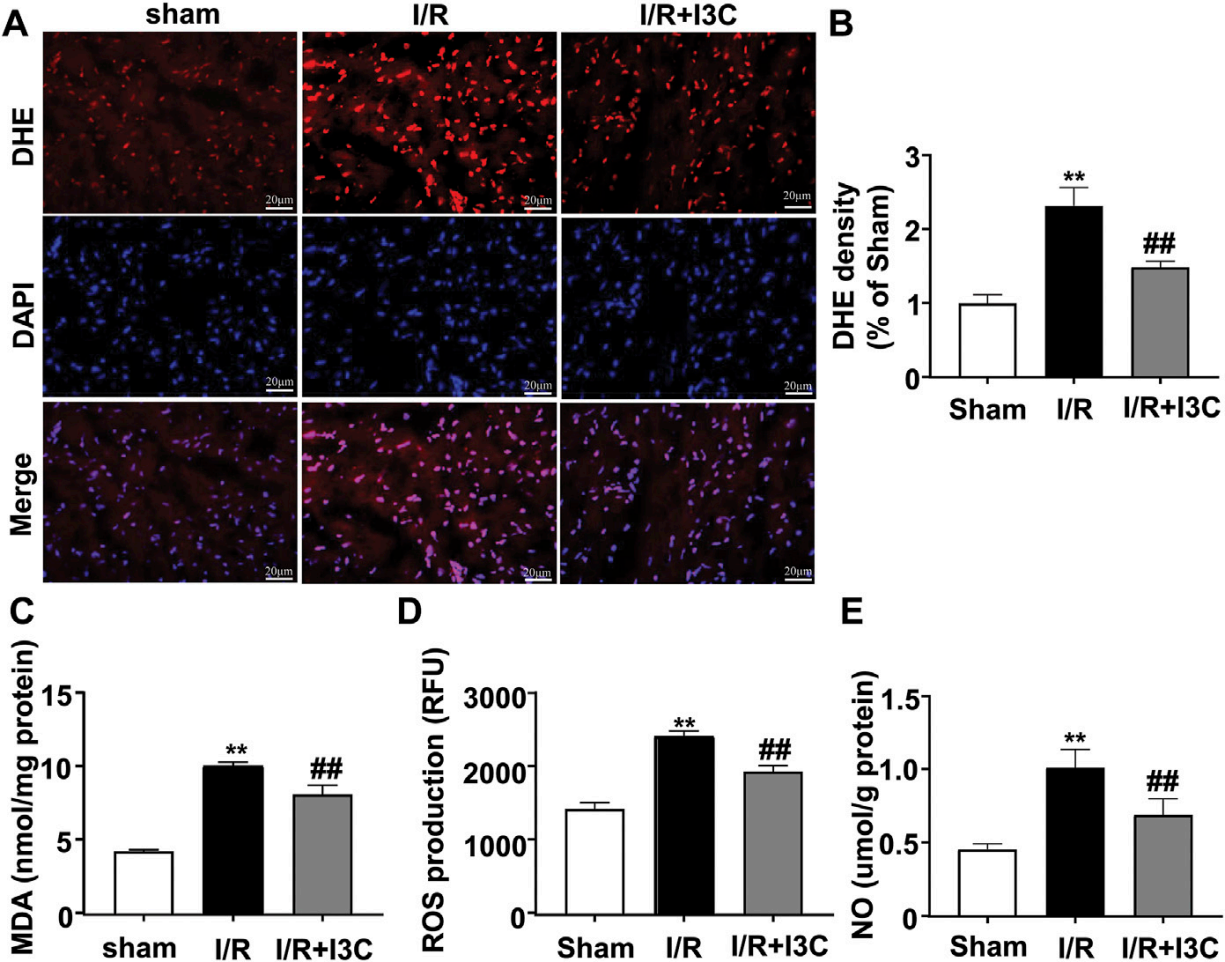
Effect of I3C on pro-oxidant stress parameters in myocardial tissue of I/R mice. **(A,B)** Representative images **(A)** and quantitative analysis **(B)** showed that I3C pretreatment (100 mg/kg) prevented the I/R-induced increase in the number of DHE-positive cells in ischemic cardiac tissue (*n* = 5, **p < 0.01 vs. sham treatment, ##*p* < 0.01 vs. I/R). **(C–E)** Quantitative analysis showed that I3C pretreatment prevented the I/R-induced increase in MDA **(C)**, ROS **(D)**, and NO **(E)** levels in ischemic heart tissue (*n* = 5, **p < 0.01 vs. sham treatment, ##*p* < 0.01 vs. I/R). Scale bars: 20 μm. Data are expressed as mean ± SEM.

**FIGURE 4:**
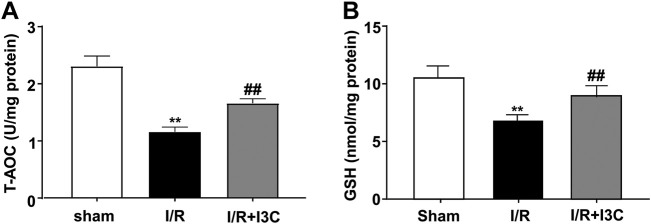
Effect of I3C on antioxidant stress parameters in myocardial tissue of I/R mice. **(A,B)** Quantitative analysis showed that the I3C pretreatment (100 mg/kg) prevented I/R-induced decrease in T-AOC **(A)** and GSH **(B)** levels in ischemic cardiac tissue (*n* = 5, **p < 0.01 vs. sham treatment, ##*p* < 0.01 vs. I/R). Data are expressed as mean ± SEM.

To investigate the role of I3C in I/R-induced oxidative stress in the ischemic heart, cardiac tissues from mice treated with vehicle, I3C, or I/R were stained with a fluorescent marker for ROS, DHE. Numerous DHE-positive cells were observed in the ischemic heart tissue of I/R mice, which were significantly reduced markedly by pretreatment with I3C (100 mg/kg, Figures 3A, B, F_2,12_ = 110.10, *p* < 0.001). In addition, it was found that I3C pretreatment also reduced the I/R-induced increases in MDA (Figure 3C, F_2,12_ = 277.80, *p* < 0.001), ROS (Figure 3D, F_2,12_ = 34.27, *p* < 0.001), NO (Figure 3E, F_2,12_ = 53.10, *p* < 0.001) levels, as well as the I/R-induced decreases in T-AOC (Figure 4A, F_2,12_ = 110.30, *p* < 0.001) and GSH (Figure 4B, F_2,12_ = 21.49, *p* < 0.001) levels in ischemic cardiac tissues in mice.

### Administration of I3C Prevented the I/R-Induced Inflammatory Responses in Myocardial Tissues

**FIGURE 5:**
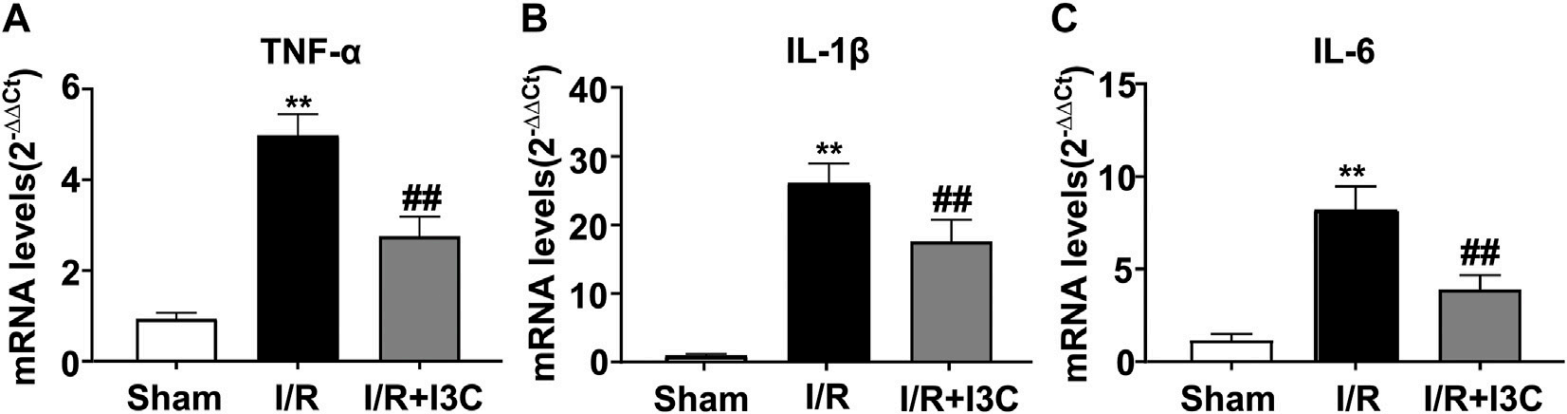
Effect of I3C on inflammatory parameters in myocardial tissue of I/R mice. **(A–C)** Quantitative analysis showed that I3C pretreatment (100 mg/kg) prevented the I/R-induced increase in the expression levels of TNF-α **(A)**, IL-1β **(B)**, and IL-6 **(C)** in ischemic cardiac tissue (*n* = 8, **p < 0.01 vs. sham treatment, ##*p* < 0.01 vs. I/R). Data are expressed as mean ± SEM.

Because inflammation is a crucial component of the pathogenesis of myocardial I/R injury, we next investigated whether administration of I3C could affect the inflammatory responses in ischemic heart tissues of I/R mice by detecting the changes in the expression levels of pro-inflammatory cytokine mRNA. The results showed that I3C pretreatment caused a remarkable decrease in the expression levels of TNF-α (Figure 5A, F_2,21_ = 315.00, *p* < 0.001), IL-1β (Figure 5B, F_2,21_ = 212.00, *p* < 0.001), and IL-6 (Figure 5C, F_2,21_ = 104.90, *p* < 0.001) mRNA in ischemic heart tissue of I/R mice.

## Discussion

Ischemia in the heart can lead to damage of the myocardium, and reperfusion can paradoxically exacerbate this damage, which is called reperfusion injury. The mechanisms contributing to the pathogenesis of ischemia/reperfusion injury are multifactorial and very complex (Lejay et al., 2016). In this study, we used a myocardial ischemia-reperfusion model to investigate the pharmacological effects and mechanisms of I3C administration on myocardial I/R injury in mice. Our results showed that I3C pretreatment significantly reduced myocardial infarction area, decreased serum LDH and CK activities, increased EF and FS, and protected cardiac functions in cardiac I/R-treated mice. These results indicated that I3C could be a potential drug for the treatment of I/R injury to the heart and provided further evidence for the cardioprotective effects of I3C.

It is known that the stimulus of ischemia-reperfusion can lead to increased oxidative stress, proinflammatory responses, and apoptosis of cardiomyocytes in ischemic heart tissue (Jose Corbalan et al., 2016). However, exactly how I3C prevents cardiac ischemia/reperfusion injury remains unclear. AhR, a ligand-dependent transcription factor that can be activated by various ligands and has immunomodulatory activities, is a potential target of I3C (Shinde et al., 2018; Khan and Langmann, 2020). Inactive AhR is mainly localized in the cytoplasm. Upon binding a ligand, it detaches from its chaperones and disperses to the nucleus, where it contributes to the maintenance of cellular homeostasis by regulating gene transcription (Larigot et al., 2018). I3C pretreatment has been shown to effectively reduce LPS-induced expression of IL-1β and IL-6 genes in microglia through activation of the AhR pathway (Khan and Langmann, 2020). In this study, we found that I3C pretreatment could prevent the I/R-induced increase in TNF-α, IL-1β, and IL-6 mRNA expression in ischemic heart tissue of mice. This finding was in some agreement with previous *in vitro* results: I3C treatment was found to reduce the hypoxia-induced increase in TNF-α, IL-1β and IL-6 mRNA expression in cultured H9c2 cells (Liang et al., 2017). Here, we speculated that I3C pretreatment in cardiac I/R models used here likely exerted anti-inflammatory functions through activation of the AhR pathway, which should be further confirmed by future studies.

I/R may not only trigger the production of high levels of proinflammatory responses, but also promote the production of free radicals in ischemic cardiac tissue (Chen et al., 1991), which likely promotes the release of intracellular components such as lysosomal enzymes, thereby triggering oxidative stress and damaging the cell membrane (Kako, 1987). NO is a small radical molecule produced by inducible nitric oxide synthase (iNOS). It has numerous physiological functions, including regulation of vascular relaxation, neurotransmission, oxidative stress, and inflammation (Zhao et al., 1996). The pathologically elevated pro-inflammatory cytokines can promote the production of NO by stimulating iNOS synthesis (Davis et al., 2001), and the overproduced NO further promotes the progression of inflammation (Hofseth, 2008). In a previous study by Khan and Langmann (2020), I3C treatment was shown to suppress the LPS-induced increase in iNOS expression and NO secretion, which subsequently contributes to the amelioration of microglial phagocytosis and migration in a manner that is partially dependent on activation of the AhR pathway. It has also been reported that I3C supplementation can attenuate DOX-induced pro-inflammatory responses by downregulating the expression of iNOS in mice (Hajra et al., 2018). *In vitro* studies have shown that I3C may be a free radical scavenger (Arnao et al., 1996). Similarly, our studies have shown that pretreatment with I3C could prevent the pathological increase in NO levels in ischemic heart tissue. This finding indicates that I3C pretreatment can prevent the ischemic heart damage by reducing the production of NO.

In addition to pathologically increased NO, the overproduction of ROS, which normally occurs during ischemia and is further exacerbated by reperfusion, also plays a key role in the development of oxidative stress during cardiac ischemia (Shilo et al., 2021). The increased ROS, together with NO, increases the production of pro-inflammatory cytokines and causes oxido-nitrosative stress, which leads to oxidative stress and cardiac dysfunction (Rawdin et al., 2013). Our results showed that I3C pretreatment prevented the I/R-induced increase in MDA and ROS levels in ischemic heart tissue of mice, which is consistent to some extent with a previous finding that I3C supplementation can inhibit lipid peroxidation (Arnao et al., 1996) and reduce production of ROS induced by microvascular dysregulation (Ampofo et al., 2018). Thus, it can be concluded that I3C supplementation is able to alleviate oxidative stress in the ischemic heart tissue of I/R mice.

Ischemia/reperfusion not only leads to high oxidative stress, but also can induce deleterious apoptosis of cardiomyocytes in ischemic heart tissue (Gottlieb and Engler, 1999). Caspase family proteins play a key role in the pathogenesis of cellular apoptosis in ischemic pathogenesis of the heart (McIlwain et al., 2013). In a previous study, treatment with I3C was found to improve cardiac function in a rat myocardial infarction model by reducing the expression of apoptotic markers such as cytochrome C, caspase-9, and caspase-3 (Ramakrishna and Krishnamurthy, 2022). Our results show that pretreatment with I3C simultaneously increased the expression levels of Bcl-2 and decreased the expression levels of Bax, caspase-3, and caspase-9, thereby contributing to the amelioration of cellular apoptosis in ischemic heart tissue. Myocardial apoptosis may also be mediated by many other signaling molecules, such as NF-κB and c-Jun N-terminal kinase (JNK) (Wu et al., 2016). It has been reported that I3C treatment can exert anti-inflammatory and anti-apoptotic effects by reducing the transcriptional activities of NF-κB in I/R-induced injury (Ampofo et al., 2017) and prevent hypoxia-induced phosphorylation of JNK in H9c2 cells (Liang et al., 2017), suggesting that I3C has cardioprotective effects likely through reducing NF-κB and JNK signaling. The exact mechanisms underlying the regulation of NF-κB and JNK by I3C require further investigation in future studies.

## Conclusion

Our results provide scientific evidence that pretreatment with I3C can prevent MIRI-induced cardiac injury through its anti-apoptotic, antioxidant, and anti-inflammatory effects. I3C could be developed as a potential drug for the treatment of I/R damage to the myaocardium. However, the present study only examined the cardioprotective effects of I3C in models of cardiac ischemia/reperfusion *in vivo*. Whether I3C has cellular protective effects in cardiac ischemia/reperfusion *in vitro* remains to be determined. This is also a major limitation for our current studies. We recommend that future studies should focus on the following: 1) investigating the specific targets and mechanisms for the cardioprotective effects of I3C in cardiac I/R models under *in vivo* conditions, 2) further clarifying the contributions of different cell types, such as cardiomyocytes and fibroblasts, in cardiac tissues to the cardioprotective effects of I3C in cardiac I/R.

## Data Availability

The original contributions presented in the study are included in the article/supplementary material further inquiries can be directed to the corresponding authors.
